# SARS-CoV-2, myocardial injury and inflammation: insights from a large clinical and autopsy study

**DOI:** 10.1007/s00392-021-01910-2

**Published:** 2021-07-19

**Authors:** Matteo Dal Ferro, Rossana Bussani, Alessia Paldino, Vincenzo Nuzzi, Chiara Collesi, Lorena Zentilin, Edoardo Schneider, Ricardo Correa, Furio Silvestri, Serena Zacchigna, Mauro Giacca, Marco Metra, Marco Merlo, Gianfranco Sinagra

**Affiliations:** 1 Cardiothoracovascular Department, Azienda Sanitaria Universitaria Giuliano Isontina (ASUGI), University of Trieste, Via Valdoni 7, 34123 Trieste, Italy; 2 International Centre for Genetic Engineering and Biotechnology (ICGEB), Trieste, Italy; 3 Institute of Pathological Anatomy, Azienda Sanitaria Universitaria Giuliano Isontina (ASUGI), University of Trieste, Trieste, Italy; 4 School of Cardiovascular Medicine and Sciences, King’s College London, British Heart Foundation Centre of Research Excellence, London, SE5 9NU UK; 5 CardiologyASST Spedali Civili di Brescia and Department of Medical and Surgical Specialties, Radiological Sciences and Public Health, University of Brescia, Brescia, Italy; 6 Department of Medical, Surgical and Health Sciences, University of Trieste, Trieste, Italy

**Keywords:** COVID-19, Myocarditis, Cardiac autopsy study, Necrosis, Myocardial injury

## Abstract

**Objective:**

Despite growing evidence about myocardial injury in hospitalized COronaVIrus Disease 2019 (COVID-19) patients, the mechanism behind this injury is only poorly understood and little is known about its association with SARS-CoV-2-mediated myocarditis. Furthermore, definite evidence of the presence and role of SARS-CoV-2 in cardiomyocytes in the clinical scenario is still lacking.

**Methods:**

We histologically characterized myocardial tissue of 40 patients deceased with severe SARS-CoV-2 infection during the first wave of the pandemic. Clinical data were also recorded and analyzed. In case of findings supportive of myocardial inflammation, histological analysis was complemented by RT-PCR and immunohistochemistry for SARS-CoV-2 viral antigens and in situ RNA hybridization for the detection of viral genomes.

**Results:**

Both chronic and acute myocardial damage was invariably present, correlating with the age and comorbidities of our population. Myocarditis of overt entity was found in one case (2.5%). SARS-CoV-2 genome was not found in the cardiomyocytes of the patient with myocarditis, while it was focally and negligibly present in cardiomyocytes of patients with known viral persistence in the lungs and no signs of myocardial inflammation. The presence of myocardial injury was not associated with myocardial inflammatory infiltrates.

**Conclusions:**

In this autopsy cohort of COVID-19 patients, myocarditis is rarely found and not associated with SARS-CoV-2 presence in cardiomyocytes. Chronic and acute forms of myocardial damage are constantly found and correlate with the severity of COVID-19 disease and pre-existing comorbidities.

**Graphic abstract:**

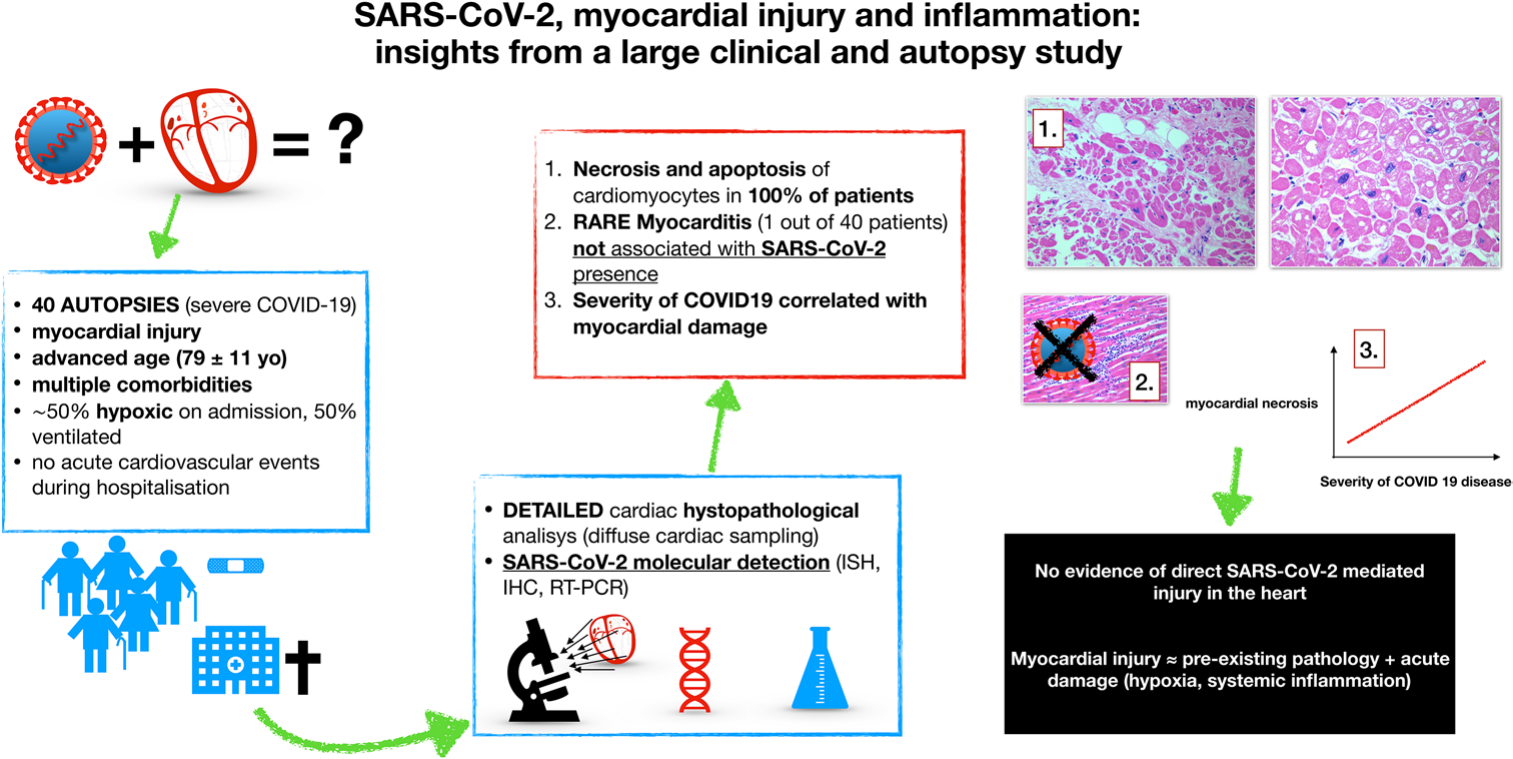

**Supplementary Information:**

The online version contains supplementary material available at 10.1007/s00392-021-01910-2.

## Introduction

Viruses are known as the most prevalent cause of myocarditis [1], and during previous viral outbreaks, which to date were mostly due to influenza A and B cases, autopsy series reported myocarditis prevalence ranging from 22 to 34% [2, 3].

During actual pandemic, in patients hospitalized for Corona Virus Disease 2019 (COVID-19), a myocardial injury (defined as concentrations of troponin in the blood above normal levels [4]), is commonly observed, involves two out of five hospitalized patients [5, 6], correlates with pre-existing cardiac morbidity and is strongly associated with adverse prognosis [7, 8].

Together with myocardial injury, a large spectrum of cardiovascular manifestations has been described in COVID-19 [9, 10], including myocarditis, suggesting a direct tropism of severe acute respiratory syndrome coronavirus 2 (SARS-CoV-2) for the heart. This hypothesis is supported by multiple lines of evidence: cardiomyocytes (CMs), pericytes and cardiac endothelial cells strongly express the SARS-CoV-2 angiotensin-converting enzyme 2 (ACE-2) receptor [11] and in vitro studies demonstrate that SARS-CoV-2 infects CMs derived from induced pluripotent stem cells (iPSC), exerting a cytotoxic effect [12].

Furthermore, anecdotal reports have identified putative viral particles in endomyocardial biopsies of patients with COVID-19 and acute myocarditis [9, 12], while some autopsy studies [13] documented the presence of SARS-CoV-2 genome in heart samples in approximately 60% of cases.

However, in the clinical arena, the prevalence of clinically relevant myocarditis is relatively low, and a definite evidence of SARS-CoV-2 infection of CMs, with or without associated cardiac inflammation, is still poorly documented [13–15]. Hospitalized patients in fact frequently suffers from pre-existing cardiac morbidities and are frequently exposed to prolonged hypoxia and sustained systemic inflammation [16]. All these elements may by themselves represent alternative causes of myocardial injury. To obtain a deeper insight into the mechanism behind SARS-CoV-2-induced myocardial injury, and to investigate its eventual association with virus-mediated myocardial inflammation, we performed a detailed histological and molecular analysis on heart samples from a previously published autopsy series of patients deceased of respiratory failure due to COVID-19 [14].

## Methods

### Study population

Forty consecutive autopsies of patients deceased of COVID-19 during the first wave of the pandemic in different departments of University Hospital of Trieste, Italy, were included in this study. All available clinical data at the admission and during hospitalization were collected.

The same population was recently published [14]: 40 out of 41 patients included in the previously published series have been included for the present detailed cardiac histopathological analysis. All patients scored positive for SARS-CoV-2 by RT-PCR tests on nasopharyngeal swab and presented symptoms (fever, cough and dyspnea) and imaging data indicative of interstitial pneumonia related to COVID-19 disease. Abnormal serum troponin level was defined according to manufacturer’s indication (Access hsTnI assay, Beckman Coulter Inc., Brea, CA, USA: above 18 ng/mL).

Histological analysis was performed by expert technicians and pathologists at the Pathology Unit of Trieste University Hospital. The same pathologists analyzed all samples considered in this study, excluding operator-dependent biases.

### Autopsy and histological studies

17 out of 40 autopsy studies were mini-invasive: the heart was not completely removed from the body, and only 4–6 specimens were sampled. For all the other studies, the number of specimen per heart varied between 15 and 30. Each patient was identified with a different progressive number (mini-invasive autopsy: from n°10 to n°26; complete autopsy: n°165, 197, 207, 210, 216, 235, 241, 256, 257, 262, 272, 276, 277, 285, 288, 300, 308, 314, 327, 354, 402, 445, 448).

Pneumonia severity score was pathologically assessed as previously described [14]: 1, multiple micro-foci of modest inflammation; 2, multiple micro-foci of moderate inflammation; 3, diffuse moderate inflammation; 4, diffuse severe inflammation; 5, massive inflammation with loss of lung architecture.

Pathological diagnosis of 1) ischemic cardiomyopathy was defined by the presence of stenosis > 75% of one or more coronary arteries, 2) hypoxic cardiomyopathy by the presence of pathological cardiac signs of pulmonary hypertension associated with chronic pulmonary disease, 3) hypertensive heart disease by the presence of cardiac hypertrophy and dilatation with increase of myocardial weight in the absence of valvular disease, 4) Valvular cardiomyopathy when severe valvular disease, or multiple moderate valvular disease was found.

Samples were fixed in 10% formalin for at least 50 h and then embedded in paraffin.

Myocarditis was defined according to Dallas Criteria as the presence of inflammatory infiltrate associated with myocyte’s injury [22, 23]. The degree of myocardial fibrosis and hypertrophy was assessed on a semi-quantitative scale: Grade 0, none; Grade 1, mild (< 10% of myocardial area); Grade 2, moderate (10–25% of myocardial area); Grade 3, severe (25–50% of myocardial area); Grade 4, massive (> 50% of myocardial area).

One-micron sections were processed for the following histological and immunohistochemistry (IHC) staining: Hematoxylin–Eosin, PAS (ABPAS), Congo-Red; antibodies against Annexin V (Novusbio H00000308-M01) was tested to confirm apoptosis, and against SARS-CoV-2 Spike protein (GeneTex GTX632604) was tested in selected heart specimens of patients in which myocarditis was identified at histological analysis. Infiltrates were further characterized by immunohistochemical staining for CD45, CD4, CD8 and CD16, performed on an automated staining device. Antigen retrieval techniques and antibody pretreatment were performed according to the manufacturer’s specifications. Images were acquired using a Leica DM2700 M light microscope.

In situ hybridization (ISH) was performed using locked nucleic acid (LNA) probes for U6 snRNA (miRCURY LNA Detection probe, Qiagen, cat. no. YD00699002) as previously described [14] and SARS-CoV-2 RNA, designed to target the sense strand of ORF1ab and Spike regions of the viral genome. A scrambled sequence probe (YD00699004) was used as a control. Experiments were performed using a dedicated ISH kit for formalin-fixed paraffin-embedded (FFPE) tissues (Qiagen) according to the manufacturer’s protocol. Briefly, FFPE tissue slides were deparaffinized in xylene, treated with proteinase-K (15 mg/ml) for 5 min at 37 °C and incubated with either SARS-CoV-2 (40 nM) or U6 probes (2 nM) for one hour at 54 °C in a hybridizer. After washing with SSC buffer, the presence of SARS-CoV-2 RNA was detected using an anti-DIG alkaline phosphatase (AP) antibody (1:500) (Roche Diagnostics) supplemented with sheep serum (Jackson Immunoresearch) and bovine serum albumin (BSA). Hybridization was detected by adding NBT-BCIP substrate (Roche Diagnostics). Nuclei were counterstained with nuclear fast red.

### RNA extraction from fixed-formaldehyde paraffin-embedded tissues and qRT-PCR

RNA was extracted from 8 µm sections, which were deparaffinized and digested. RNA extraction was performed with Trizol (Invitrogen), following manufacturer’s instructions with some modifications.

The quality of total RNA was assessed by measuring the expression of GAPDH. As controls, we used a known positive sample, subjected to the same RNA extraction procedure, and a SARS-CoV-2 MULTITARGET RNA. Detection of SARS-CoV-2 was performed using commercially available primers and probes (Eurofins Genomics) for N and ORF1ab genes.

### Statistical analysis

Data on a continuous scale are reported as mean and standard deviation or median and interquartile range as appropriate; discrete parameters are reported as counts and percentages.

Correlations were graded by the coefficient of determination *R*^2^ (Prism Software, GraphPad, version 5).

## Results

### Study population

Clinical characteristics of patients are summarized in Table 1. Mean age of patients was 79 years, 55% were males. All suffered of COVID-19 pneumonia, and 47% of them presented with O2 Saturation < 90% in ambient air on admission. Pneumonia was moderate to severe in 75% of patients, and 50% of them were subjected to ventilation therapy (mechanical and/or non-invasive). CRP levels on admission were significantly elevated. History of hypertension, heart failure, diabetes, chronic kidney disease involved 37, 32, 24 and 20% of patients, respectively. Only 17.5% of the patients was on oral anticoagulant therapy at admission (median INR 1.32 (1.08–1.68)), while about 30% were treated with heparins during hospitalization. One-third of the patients was affected by ischemic cardiomyopathy, including previous myocardial infarction and/or myocardial revascularization. Half of the patients had multiple extracardiac comorbidities, including cancer and neurological diseases.

**Table 1:** Clinical characteristics of the study population

Clinical characteristics	
Age, years	79 ± 11
Hospitalization, days	17 (8–27)
Time from symptom onset to hospitalization, days	4 (1–8)
O_2_ Saturation < 90% on ambient air at admission, no.% (36 pts)	17 (47.2)
CRP, mg/dl	159 (60–239)
d-dimer, LFEU (9 pts)	1.9 (1.1–15.7)
NTproBNP, ng/ml (17 pts)	7090 (1604–22,239)
EF, % (7 pts)	50 (12)
Troponin ng/ml (19 pts)	55 (20–490)
INR at admission (33 pts)	1.32 (1.08 –1.68)
OAC therapy at admission, no.%	7 (17.5)
Acetylsalicylic acid, no. %	15 (37)
ACEi/ARB, no. %	12 (29)
Hypertension, no. %	15 (37)
Diabetes, no. %	10 (24)
Heart failure, no. %	13 (32,5)
Chronic kidney disease, no. %	8 (20)
Heparin/LMWH/anti-k drugs during Hospitalization, no. %	11 (27)
Intra-hospital PE, no. %	3 (7)
Corticosteroid use during Hospitalization, no. %	3 (7)
Pneumonia severity	Grade 1: 2 (5) Grade 2: 8 (20) Grade 3: 9 (22) Grade 4: 12 (29) Grade 5: 10 (24)
ICU stay, no.%	8 (20)
Max ventilatory support	Mech: 8 (20) NIV: 12 (30)
N° patients with extracardiac comorbidities	0: 2(5) 1: 18 (45) 2: 15 (37.4) 3: 4 (10) 4: 1 (2.5)

Continuous values are expressed by median (interquartile range)

*ICU* Intensive Care Unit; *CRP* C Reactive Protein; *Mech* mechanical Ventilation *NIV* Non-Invasive Ventilation; *INR* International Normalized Ratio, *OAC* oral anticoagulant therapy (both Vitamin K Antagonists and DOACs), *PE* Pulmonary Embolism; *ACEi* Ace-Inhibitors; *ARB* Angiotensin Receptor Blockers, *LMWH* Low molecular weight heparin

Troponin abundance was available for 19 patients and in all, but 1 cases, it was consistent with myocardial injury (Median value: 55 ng/L, IQR 20–490). Echocardiographic data were available for seven patients and showed left ventricular systolic dysfunction (i.e. left ventricular ejection fraction < 50%) in one case, who had known history of previous myocardial infarction. With the exception of one case (frequent ventricular arrhythmias requiring lidocaine infusion in the patient with previous myocardial infarction and severe left ventricular dysfunction), clinical course of these patients was free from acute cardiovascular events.

### Detailed cardiac histological analysis reveals heterogeneous, nonspecific, acute and chronic forms of myocardial damage and rarely detects overt myocarditis

**Fig. 1:**
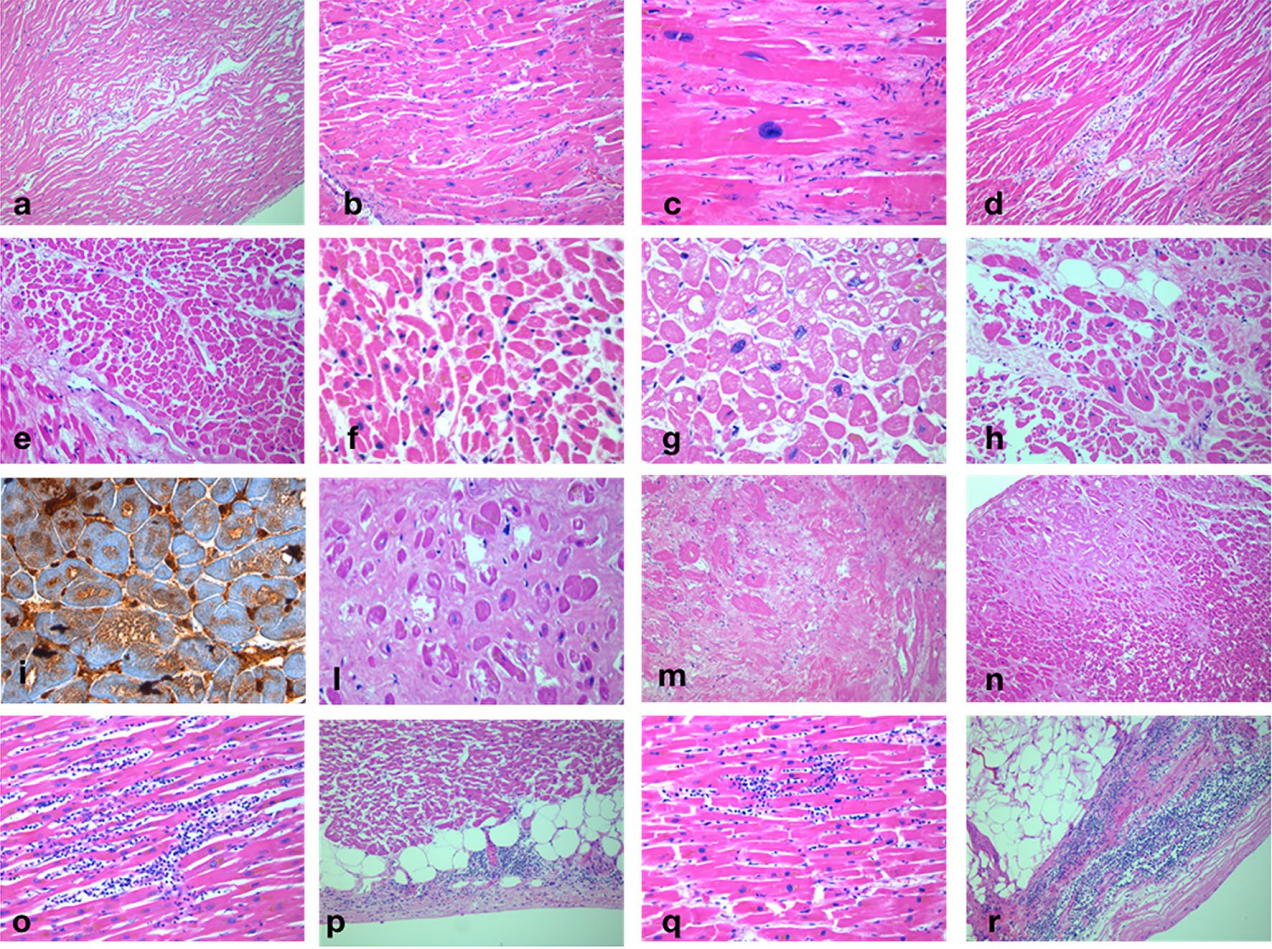
Hematoxylin–Eosin staining of cardiac tissue showing different forms of damage. **a** Sample 210, wavy myocells (× 10), **b** Sample 272, CM hypertrophy (× 10), **c** Sample 272, CM hypertrophy (× 40), **d** Sample 197, Interstitial fibrosis (× 20), **e** Sample 165, CM apoptosis (× 20), **f** Sample 241, CM apoptosis e necrobiosis (× 40), **g** Sample 300, CM necrosis, apoptosis and hypertrophy (× 40), **h** Sample 207, CM necrosis, apoptosis (× 20), **I** Sample 197, IHC for Annexin V (× 40), **l** Sample 285, Substitutive fibrosis (× 10), **m** Sample 235, severe substitutive fibrosis (× 10), **n** Sample 288, amyloidosis (× 10), **o** Sample 314, overt lymphocytic myocarditis (× 20), **p** Sample 354, lymphocytic epicarditis (× 10), **q** Sample 262, spotty lymphocytic myocarditis (× 20), **r** Sample 285, lymphocytic epicarditis (× 10)

**Fig. 2:**
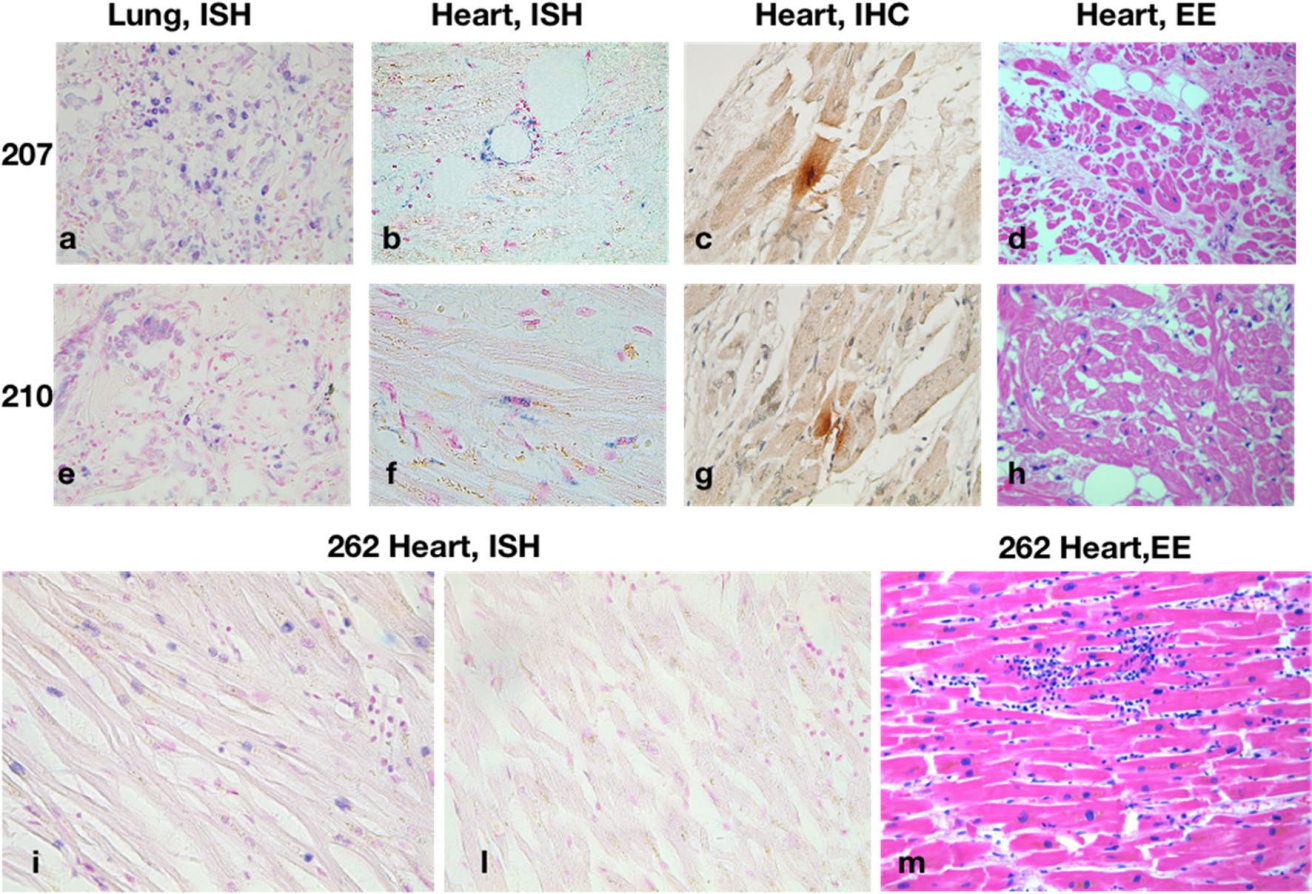
Cardiac ISH of SARS-CoV-2 RNA and IHC against SARS-CoV-2 Spike protein on patients with no myocarditis and known viral persistence in the lungs (n°207 and 210) and in a representative patient with myocarditis (n°262). **a** Sample 207, Probe SARS-CoV-2 Rna; **b** Sample 207, Probe SARS-CoV-2 Rna; **c** Sample 207, Antibody SARS-CoV-2 Spike; **d** Sample 207, hematoxylin–eosin. **e** Sample 210, Probe SARS-CoV-2 Rna; **f** Sample 210, Probe SARS-CoV-2 Rna; **g** Sample 210, Antibody: SARS-CoV-2 Spike; **h** Sample 210, hematoxylin–eosin. **i** Sample 262, Probe control U6; **l** Sample 262, Probe SARS-CoV-2 Rna; **m** Sample 262, hematoxylin–eosin

Pathological findings are summarized in Table 2. Macroscopic examination of the heart was available for 23 cases. Ischemic cardiomyopathy resulted as the most prevalent diagnosis (14 samples, 60%), followed by hypoxic cardiomyopathy (eight samples, 34%). These diagnoses co-existed in two patients. Histopathological analysis was available for all cases. All the 40 hearts presented features of tissue damage and/or age-related degeneration. In detail, myocells with a wavy appearance, indicative of myocardial disarray, were identified in 36 samples, while fibrosis, with different severity or localization, was present in all samples (Fig. 1: panel a–d, i, l). CM necrosis, apoptosis, hypertrophy, and myofibril attenuation were also identified in all samples. The distribution of tissue degeneration was non-coronaric, mostly multifocal (Fig. 1: panel e–h, m, Fig. 2: panel d, h, and Supplementary Fig. 1). Cardiac amyloidosis was evident in 30% of samples and were all linked to wild-type transthyretin deposition (Fig. 1: panel n).

**Table 2:** Pathological and Histopathological findings in the study population

Pathological (n° 23) and Histopathological (n°40) cardiac findings	
Hypoxic heart disease, no. %	8 (32)
Ischemic heart disease, no %	12 (55)
Hypertensive heart disease, no. %	5 (21)
Heart weight, g	523 (142)
Microcirculatory pulmonary thrombosis, no. %	29 (71)
Hypertrophy, no. %	Grade 1: 5 (12) Grade 2: 25 (61) Grade 3: 11 (27)
Fibrosis, no. %	Grade 1: 10 (24) Grade 2: 18 (44) Grade 3: 10 (24) Grade 4: 3 (7)
Wavy fibers, no. %	Absent: 4 (10) Focal: 4 (10) Diffuse: 32 (80)
Apoptosis, no. %	Focal: 5 (12) Subendocardic: 2 (5) Multifocal: 19 (46) Diffuse, severe: 14 (34)
Necrosis, no. %	Focal: 8 (19) Subendocardic: 1 (2) Multifocal: 20 (49) Diffuse, severe: 11 (27)
Epicarditis, no. %	12 (29)
Myocarditis 3 + /1 + , no. %	1 (2,5)/2 (5)
Amyloidosis, no. %	11 (27)
Microcirculatory alterations, no. %	Thrombosis: 0 Diffuse Fibrosis: 2 (5)

**Fig. 3:**
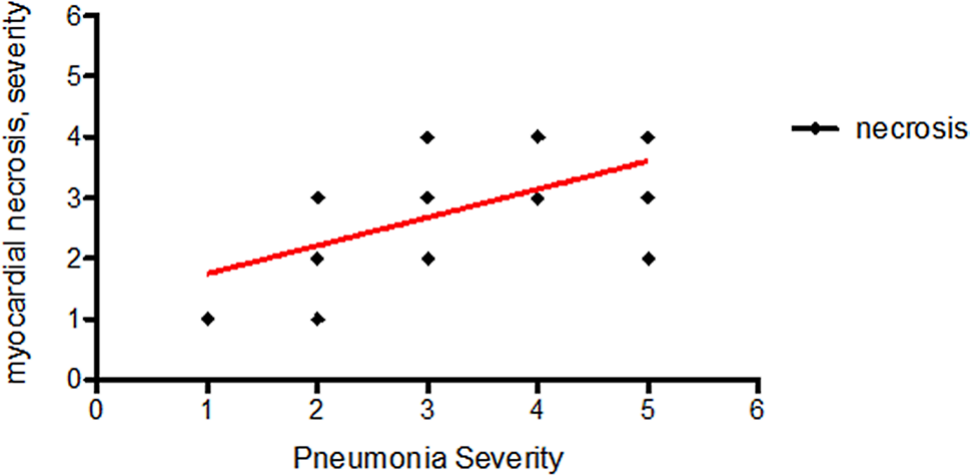
Correlation between Pneumonia severity (Grade 1–5) and Myocardial necrosis (Grade 1-focal- to 4 -diffuse, severe-): *R*^2^ = 0.37; *p* < 0.0001

Inflammatory infiltrate was carefully assessed: notably, mild-to-moderate lymphocytic epicarditis was identified in about one-third of cases. Overt lymphocytic myocarditis was evident in one case (n°314), and single or multiple foci of localized myocarditis characterized two additional samples (n°354, 262), with different predominance of CD4, CD8 and CD16 lymphocytes. (Fig. 1: panel n–r, Supplementary Fig. 2). All patients with available serum troponin dosage on admission (*n* = 19) showed moderate-to-severe myocardial damage, including both acute (CM necrosis, apoptosis, wavy fibers) and chronic (fibrosis) features. The vast majority of these patients (*n* = 15) did not show any inflammatory infiltrate, while the remaining four showed mild epicarditis. A statistically significant correlation was found between severity of pneumonia and severity of myocardial necrosis (*R*^2^ = 0.37; Fig. 3).

### No molecular evidence for SARS-CoV-2 presence in hearts showing myocarditis

To investigate whether infection by SARS-CoV-2 was associated with histological evidence of myocarditis, the presence of viral RNA in selected heart samples of patients with myocarditis (n°314, 354, 262) was analyzed by ISH and RT-PCR. Expression of the Spike protein was evaluated by IHC. All these analyses showed no sign of viral presence in CMs (Sample 262: Fig. 2 i, l, m, data not shown for sample 314–354, and for negative RT-PCR). Detailed clinical characteristics of these patients can be found in Supplementary Table 1.

We successively performed the same analysis in selected heart samples of patients (n°207, n°210) with similar length of hospitalization, no histological signs of myocardial infiltrates (Fig. 2d, h) and documented viral persistence in the lung (Fig. 2a, e). Exhaustive description of SARS-CoV-2 infection in the lungs and other organs is reported in Reference 14. RT-PCR analysis did not detect the presence of the viral genome in any sample from cases 207 and 210, while sparse cells (CMs, endothelial cells, and pericytes) scored positive by ISH and Spike IHC (Fig. 2b, f, c, g). These data further exclude the presence of a relevant amount of virus in hearts of patients with severe COVID-19, even in the case of persistent SARS-CoV-2 in the lungs.

## Discussion

In this study, we provide a detailed histological characterization of the heart in an autopsy series of 40 patients deceased from SARS-CoV-2 infection during the first wave of the pandemic in the North of Italy. Only two of them showed signs of myocarditis and only one patient was affected by overt lymphocytic myocarditis. Importantly, using RT-PCR, ISH and IHC, we did not detect viral presence in all three cases characterized by histological signs of myocardial lymphocytic infiltrate.

To date, this represents one of the largest autopsy series of patients deceased by COVID-19 and the one with the highest amounts of analyzed specimens per heart. Therefore, these findings provide exhaustive information on the relationship between SARS-CoV-2 and CMs.

Our results showed pre-existing, age-related, cardiac pathologies in all analyzed cases. CM hypertrophy and different degrees of fibrosis represent the hallmark of chronic conditions, coherently with the mean number of comorbidities in our population. The presence of pre-existing cardiac chronic damage is known to exacerbate necrotic events if a new noxa occurs. Acute forms of myocardial injury (wavy myocells and CMs necrosis and apoptosis) were in fact invariably present, and particularly severe and diffuse in all cases with known troponin release. Furthermore, nonspecific sub-epicardial lymphocytic infiltrates were frequently identified (1/3 of patients), while myocarditis, mild or moderate, characterized only three cases. Unfortunately, troponin abundance was determined only in half of our cohort, but among the patients with known troponin release, no one showed myocarditis.

Focusing on myocarditis, the prevalence in our cohort ranges from 2.5%, limited to the only overt case, to 7.5%, including also the two focal cases. This prevalence is superimposable to that reported by a recent literature review and other autopsy series [17, 22], but significantly lower in respect to previous autopsy series of patients deceased from viral pneumonia due to different viruses (e.g.: influenza A or B viruses), which ranges from 22 to 34% [2, 3]. Notably, the use of steroids was negligible in our population, and the length of hospitalization of our patients corresponds to the “second phase” of myocarditis pathogenesis, in which immunological infiltrates are supposed to be present [1].

We did not detect the viral genome or Spike protein in heart samples of patients with lymphocytic myocarditis. On the contrary, ISH and Spike protein scored occasionally positive in a few perivascular cardiac cell types, including CMs, in patients with known strong persistence of viral genome in lung specimens and no signs of myocardial infiltrate.

Other reports have described quite consistently positive PCR reactions for SARS-CoV-2 genome in heart samples [13, 18], although these data do not allow one to distinguish either productive viral infection (e.g., viral protein expression) or to identify virus-infected cells (e.g., in specific tissue cell types or circulating in the blood) [9]. To the best of our knowledge, only one single pre-print work [19] has so far reported, using RNAscope for in situ genome detection, viral presence inside CMs, in the absence of inflammatory infiltrate, in six autoptic cases of COVID-19 deaths occurred during the first week of hospitalization.

Although it is known that virus-mediated CM damage only eventually triggers an inflammatory reaction [1], the low prevalence of clinical and subclinical myocarditis in fatal cases in our and other series [22] provide strong support to the conclusion that myocarditis does not play a major role among the causes of myocardial injury in the course of COVID-19 disease. Furthermore, in our cohort, the rare cases of myocarditis were not associated with SARS-CoV-2 presence, suggesting alternative causes of myocarditis (not investigated in this work).

Apoptosis and necrosis, with non-ischemic distribution, were identified in all hearts: in the early phases of infection, SARS-CoV-2 and other viruses are known to potentially trigger several types of regulated cell death [21]. Additionally, cardiac MRI studies of unselected non-fatal COVID-19, reporting high prevalence of imaging markers of myocardial acute damage [20], may suggest a frequent subclinical cardiac involvement, potentially due to such forms of cell death. However, considering the quite prolonged length of hospitalization of our cohort, we are aware that our current knowledge on the pathogenesis of myocarditis does not support the hypothesis of a persistent, virus-mediated, acute myocardial injury in the absence of inflammatory infiltrates.

On the other hand, in our patients, histological cardiac findings may rather be nonspecific and determined by multiple alternative causes. Almost half of our patient were hypoxemic on admission, and may have suffered of additional phases of hypoxia during hospitalization, so acute hypoxic cardiomyopathy, or a combination of acute hypoxia with pre-existing pathology, along with the presence of pro-apoptotic chemokines (e.g. TNFα, from systemic circulation or locally released by infected endothelial cells) may easily lead per se to necrosis and apoptosis, as supported by the significant correlation found between the severity of pneumonia and the amount of CM death. In this view, viral dissemination during viremia, with or without occasional and transient infection of CMs, pericytes or endothelial cells, seems more reasonably a rare by-stander rather than the direct cause of CM damage.

Finally, evidence was recently provided on frequent cardiac microcirculatory thrombosis in COVID-19 fatal cases [18], associated with CM necrosis, the latter not invariably present. In our cohort, which frequently showed pulmonary microvascular thrombosis (71%, (14)), we did not detect significant microvascular abnormalities in the heart, despite diffuse histological sampling, and necrosis showed mostly a non-coronaric distribution. The reasons behind this discrepancy are unknown, although we cannot exclude that our cohort may be affected by a more advanced and prolonged disease. According to this view, in our patients, which in the majority of cases had normal coagulation tests on admission, a transient microvascular thrombosis in the heart may have contributed, in a small part, to myocardial injury and necrosis. However, from a clinical point of view, we could not document acute cardiovascular events during hospitalization, and the incidence of these events in cohorts of hospitalized patients with COVID-19 is generally really low [8] when compared to their cardiovascular risk profile.

## Conclusions

In a large autopsy series of COVID-19 deceased patients, myocarditis of at least moderate entity was identified in one out of 40 hearts, while all the hearts exhibited evidence of both pre-existing chronic and acute damage, the latter proportional with the severity of pneumonia process. Viral genome and Spike protein were not identified in CMs of patients with myocarditis. Taking all our evidence together, cardiac pathological findings do not support a direct virus-mediated injury, of any type, in the heart.

## Limitations

Our results derive from retrospective observations in unselected population without control cohort and may be strongly influenced by the comorbidities affecting our patients. ISH, RT-PCR and IHC were performed only in a limited number of patients and specimens per heart, and their divergent results in cardiomyocytes may be due to sampling error. The same limit applies in particular to RT-PCR analyses, which (1) have been performed on a limited number of paraffin embedded samples and (2) may be affected by RNA degradation, which in turn may be due to RNA degradation before tissue sampling, or to heterogenous viral RNA distribution inside the hearts (“sampling error”), or to very low number of viral genomes (under the detection threshold, which is 40 viral genomes/microgram of RNA) or, finally, to only transient virus contamination/infection. RT-PCR was performed only for SARS-CoV-2 genome and not for other common cardiotropic viruses. ISH on pulmonary tissue are only briefly mentioned in this work, as they do not represent our main focus: please refer to the paper cited at ref n° 14 for a complete description of SARS-CoV-2 infection in the lungs and other organs.

Furthermore, troponin abundance was determined only in half of the patients limiting the power of our conclusion about myocardial injury.

## Supplementary Information

Below is the link to the electronic supplementary material.

Supplementary file1 (DOCX 2726 KB)

## Data Availability

All data are available upon reasonable request.
