# Correction to: SARS‑CoV‑2, myocardial injury and inflammation: insights from a large clinical and autopsy study

**DOI:** 10.1007/s00392-021-01919-7

**Published:** 2021-08-16

**Authors:** Matteo Dal Ferro, Rossana Bussani, Alessia Paldino, Vincenzo Nuzzi, Chiara Collesi, Lorena Zentilin, Edoardo Schneider, Ricardo Correa, Furio Silvestri, Serena Zacchigna, Mauro Giacca, Marco Metra, Marco Merlo, Gianfranco Sinagra

**Affiliations:** 1 Cardiothoracovascular Department, Azienda Sanitaria Universitaria Giuliano Isontina (ASUGI), University of Trieste, Via Valdoni 7, 34123 Trieste, Italy; 2 International Centre for Genetic Engineering and Biotechnology (ICGEB), Trieste, Italy; 3 Institute of Pathological Anatomy, Azienda Sanitaria Universitaria Giuliano Isontina (ASUGI), University of Trieste, Trieste, Italy; 4 School of Cardiovascular Medicine and Sciences, King’s College London, British Heart Foundation Centre of Research Excellence, London, SE5 9NU UK; 5 CardiologyASST Spedali Civili di Brescia and Department of Medical and Surgical Specialties, Radiological Sciences and Public Health, University of Brescia, Brescia, Italy; 6 Department of Medical, Surgical and Health Sciences, University of Trieste, Trieste, Italy

## Correction to: Clinical Research in Cardiology 10.1007/s00392-021-01910-2

The original version of this article, published on July 19, 2021, contained a mistake.

The spelling of Furio Silvestri’s name was incorrect. The original article has been corrected.

