# Supplementary material for: SARS-CoV-2, myocardial injury and inflammation: insights from a large clinical and autopsy study
Source: Clin Res Cardiol. 2021 Jul 19;110(11):1822–31. doi: 10.1007/s00392-021-01910-2 (PMC8288413; doi:10.1007/s00392-021-01910-2)
Supplement: Supplementary file 1 — Supplementary file1 (DOCX 2726 KB) [file 392_2021_1910_MOESM1_ESM.docx]

**Supplementary Table 1**: clinical characteristics of patients with myocarditis (262,354 and 314) and with no myocarditis (207 and 210) but evidence of mild SarsCov2 RNA presence in the heart.

| Pa-tient | Age | Sex | Comorbidities | Initial symptoms | Time from symptoms onset until admission, days | Time from admission to deaths, days | Elevated Creatinine | Elevated Troponin | Additional respiratory pathogens | Lymphopenia | Cause of death |
| --- | --- | --- | --- | --- | --- | --- | --- | --- | --- | --- | --- |
| 262 | 82 | Male | Diabetes, previous bladder cancer | Respiratory distress, fever | 7 | 16 | No | No | - | No | Adult respiratory distress failure; viral pneumonia |
| 354 | 68 | Male | Chronic obstructive pulmonary disease, schizophrenia | Respiratory distress, fever | 10 | 59 | No | No | Pseudomonas aeruginosa pneumonia | Yes | Acute hypoxic respiratory failure; adult respiratory distress failure; co-incident viral and bacterial pneumonia |
| 314 | 89 | Female | Ischemic cardiomyopathy, hypertension, erosive gastritis | Respiratory distress, fever - | - | 28 | - | - | Bacterial pneumonia | - | Acute hypoxic respiratory failure; adult respiratory distress failure; co-incident viral and bacterial pneumonia |
| 207 | 76 | Female | Ischemic and valvular aortic, coronary bypass, aortic valve replacement, chronic heart failure | Cough, respiratory distress, fever | 3 | 21 | No | Mild | No | Yes | Adult respiratory distress failure; viral pneumonia |
| 210 | 73 | Male | - | Respiratory distress, fever | 8 | 29 | Yes | No | No | Yes | Multiple organ failure; adult respiratory distress failure; viral pneumonia |

**Supplementary Figure 1.** **Apoptosis in cardiac tissues**

**
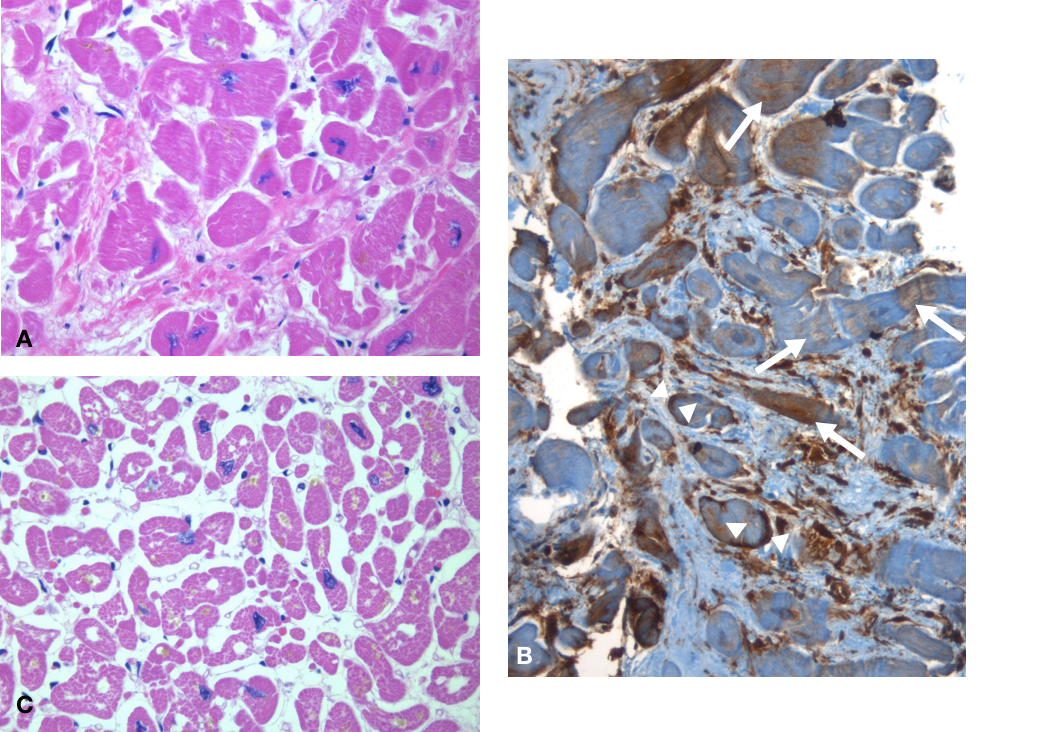
**

Panels representative of apoptosis with heavy shrinkage of the myocells and nucleus, abnormal nuclear chromatin, frequent karyorrhexis, nuclear and contractile fibers loss. Panel A): Sample 197, Hematoxylin- Eosin, apoptosis x40, B): Sample 197, IHC Annexin 5 x20; C): Sample 207, Hematoxylin- Eosin, apoptosis, x40 White arrows: cytoplasmatic-sarcolemmal signal by Annexin V antibody. White arrowheads: transversal view of the same staining.

**Supplementary Figure 2. Characterization of Lymphocytic infiltrates in myocardits**


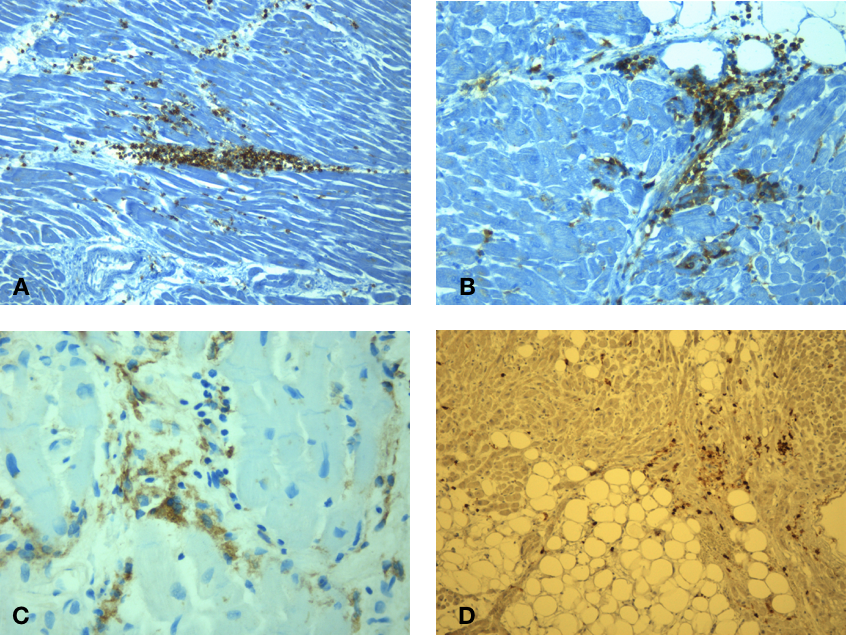


Panel A): Sample 314, CD8+ lymphocytes, x10, B): Sample 314, CD4+ lymphocytes; x20 C): Sample 354, CD16+ lymphocytes x40 D): Sample 262, CD45+ lymphocytes x10.
